# Gastrocolic Fistula in a Child Following Ingestion of Multiple Magnetic Beads

**DOI:** 10.7759/cureus.107329

**Published:** 2026-04-19

**Authors:** Jakia Nur Kheya, Kowshik Bhowmick, MST Kamrun Nahar

**Affiliations:** 1 General Surgery, Asgar Ali Hospital, Dhaka, BGD; 2 Pediatric Surgery, Asgar Ali Hospital, Dhaka, BGD; 3 Neurological Surgery, Dhaka Medical College Hospital, Dhaka, BGD

**Keywords:** gastrocolic fistula, ingested foreign body, magnetic beads, multiple magnet ingestion, emergency laparotomy

## Abstract

Foreign body ingestion is common in the pediatric population and can be challenging to manage due to delays in diagnosis. Ingestion of multiple magnets is particularly hazardous, as it can lead to pressure necrosis, perforation, and fistula formation, often necessitating urgent endoscopic or surgical intervention. We report a case of a four-year-old boy who developed a gastrocolic fistula following ingestion of multiple magnetic beads that initially remained undiagnosed. Although five beads were removed endoscopically, complete retrieval was not possible. Emergency laparotomy revealed a gastrocolic fistula between the posterior gastric antrum and the transverse colon, with the remaining magnets lodged within the fistula tract. The retained beads were removed surgically, and the gastric and colonic defects were repaired primarily. This case emphasizes that both parents and clinicians should maintain a high index of suspicion for magnet ingestion and pursue early imaging and timely intervention to prevent serious gastrointestinal complications.

## Introduction

Foreign body ingestion is common in children, with most cases resolving spontaneously [1,2]. However, ingestion of multiple magnets poses a higher risk, as their strong magnetic attraction can compress adjacent bowel loops and lead to ulceration, necrosis, perforation, and fistula formation [3,4]. These events are often unwitnessed, which may delay diagnosis and increase the risk of gastrointestinal injury [5,6]. Gastrocolic fistula is a rare but serious complication of magnet ingestion in children. Early recognition, appropriate imaging, and timely endoscopic or surgical management are important in reducing associated morbidity [7]. This report describes a rare case of gastrocolic fistula following ingestion of multiple magnetic beads in a four-year-old boy.

## Case presentation

A four-year-old boy presented to the pediatric surgery outpatient department with a history of abdominal pain and nausea for three days. The pain was sudden in onset, colicky in nature, aggravated after meals, and relieved temporarily by antispasmodic medication. His oral intake was good, and no dysphagia or drooling was reported. He had no history of fever, upper respiratory tract symptoms, chest pain, previous admissions, surgeries, or allergies. There was no history of developmental delay. He had visited local physicians several times with similar complaints over the preceding two months and had been treated conservatively with anti-ulcer and antispasmodic medications, which provided only temporary relief. 

On examination, the child was alert, interactive, and hemodynamically stable. Abdominal examination revealed a soft, non-distended abdomen with mild tenderness in the epigastric region, without guarding or rigidity.

**Figure 1:**
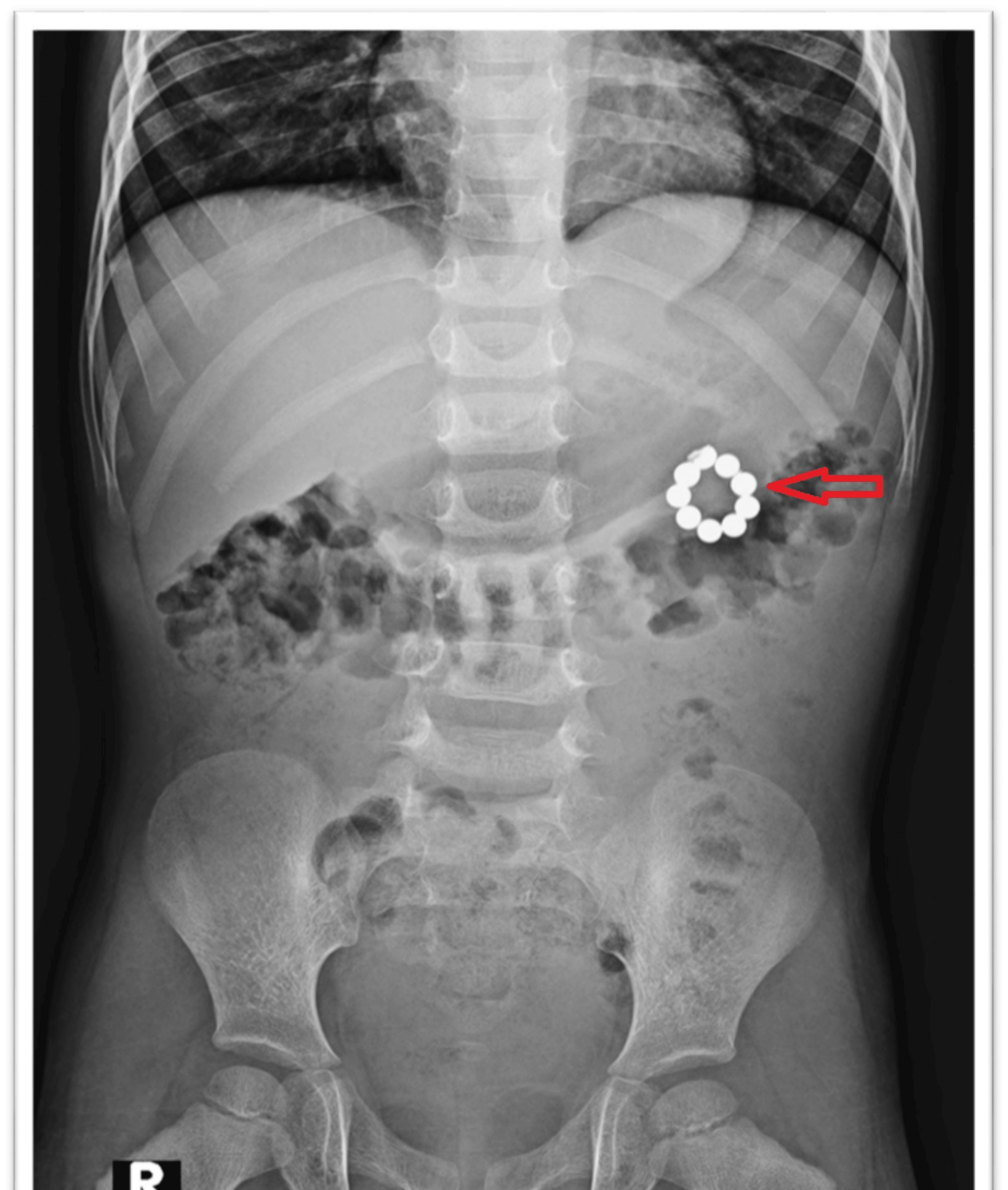
Abdominal X-ray. Plain abdominal radiograph showing multiple magnetic beads arranged in a ring-like pattern (red arrow).

**Figure 2:**
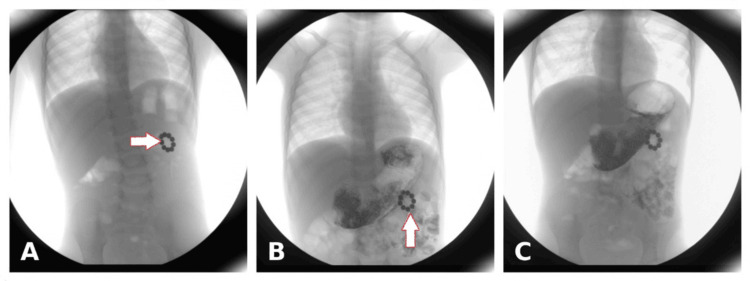
Barium meal X-ray of stomach and duodenum. (A) and (B) Barium meal X-ray of the stomach shows a circular metallic foreign body with a beaded appearance (white arrow) at the greater curvature of the stomach. (C) The duodenal cap appears normal in shape and is well opacified with contrast.

A plain abdominal radiograph demonstrated multiple radiopaque spherical foreign bodies arranged in a garland pattern over the left upper quadrant of the abdomen (Figure 1). The exact time of ingestion could not be determined as the ingestion was unwitnessed; however, the parents later identified the beads as part of a magnetic bracelet after finding two similar beads in the child's toy box. A barium meal study of the stomach and duodenum was performed on the same day to better characterize the anatomical relationship of the suspected foreign body. It revealed a circular metallic foreign body located outside the confined stomach area, causing an indentation on the greater curvature with localized mucosal thickening and irregularity (Figures 2A-2C).

**Figure 3:**
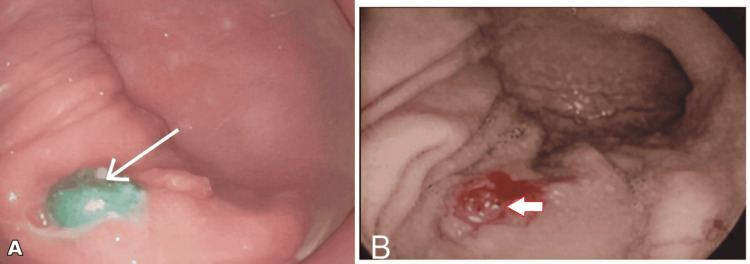
Endoscopy (stomach). (A) Endoscopic view of a magnetic bead within the stomach (white arrow). (B) Endoscopic view of magnetic beads eroding into the posterior gastric wall (white arrow) and surrounding ulceration.

These findings raised concern for possible transmural migration or fixation of the magnets across adjacent bowel segments rather than a purely intraluminal location. Consequently, a combined approach with urgent upper gastrointestinal endoscopy and preparedness for surgical exploration was planned. As part of the pre-anesthetic assessment, the child underwent laboratory investigations, including complete blood count, coagulation profile, and serum biochemical tests, all of which were within normal limits. Upper gastrointestinal endoscopy revealed multiple magnetic beads embedded within the posterior gastric wall with surrounding ulceration (Figures 3A, 3B). Five magnetic beads were successfully retrieved endoscopically. Four additional beads were seen embedded within the gastric wall and could not be removed endoscopically. Due to incomplete retrieval and concern for transmural injury, the patient underwent emergency laparotomy immediately after the endoscopic procedure on the same day.

**Figure 4:**
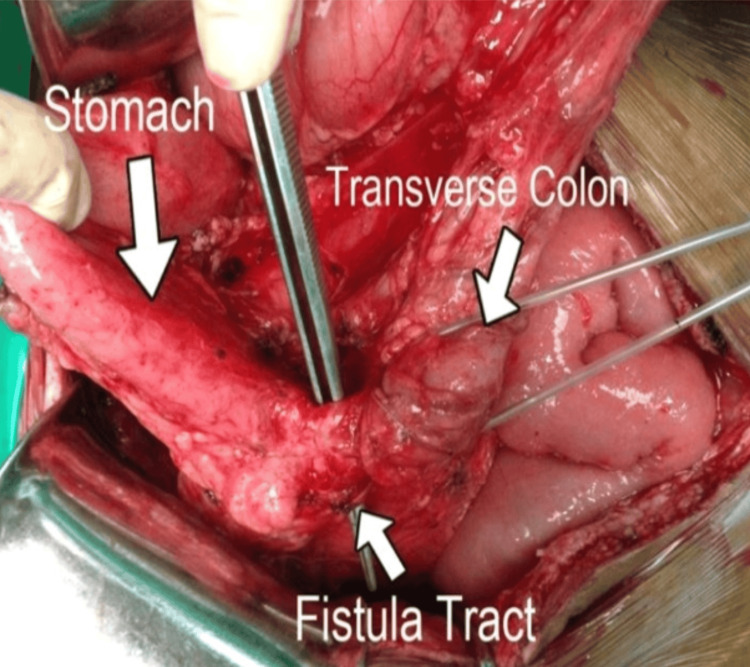
Intraoperative view of a gastrocolic fistula. This intraoperative photograph demonstrates the stomach and transverse colon, with a clearly identifiable fistula tract between them (white arrows). The tract appeared thickened with surrounding inflammatory changes and adhesions, suggestive of a chronic pathological process.

At laparotomy, the posterior wall of the gastric antrum was found densely adherent to the transverse colon, with formation of a gastrocolic fistula (Figure 4). Careful adhesiolysis was performed to separate the stomach from the colon, and the fistula tract was identified and divided. The magnets were present within the fistula tract. The tract appeared thickened and inflamed, with surrounding indurated tissue suggestive of chronic pressure necrosis. 

Following division of the fistula, a defect measuring approximately 1-1.5 cm was noted on the posterior wall of the gastric antrum, and a corresponding defect of similar size was present on the transverse colon. The remaining magnetic beads were retrieved through the colonic opening. The gastric defect was closed primarily in two layers, with an inner continuous absorbable suture for the mucosa and an outer interrupted seromuscular layer. The colonic defect was similarly repaired in two layers using absorbable sutures. The integrity of the gastric repair was assessed intraoperatively with saline instillation leak testing, which showed no evidence of leakage. After thorough peritoneal lavage, hemostasis was ensured, and the abdomen was closed in layers.

**Figure 5:**
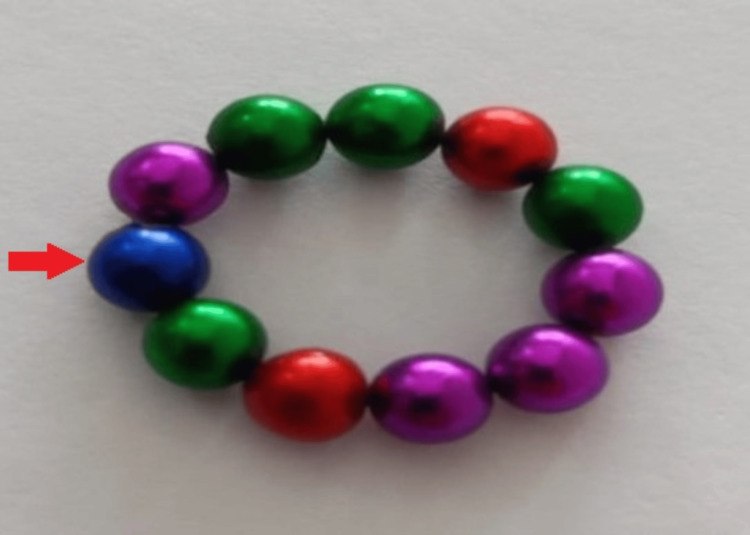
Retrieved magnetic beads in a circle. The beads are small, smooth, rounded, and multicolored, each measuring less than 4 mm in diameter (red arrow).

In total, eleven beads were identified as part of the bracelet set; nine were retrieved from the patient (five endoscopically and four surgically), and two were recovered separately by the parents. The magnetic beads were round, each measuring approximately 4 mm in diameter (Figure 5). 

The postoperative course was uneventful. The nasogastric tube was removed on postoperative day three, and oral feeding was initiated and well tolerated. The patient was discharged in stable condition on postoperative day six. Clinical follow-up at two and six weeks demonstrated complete recovery, with normal oral intake, appropriate weight gain, and regular bowel function. Abdominal examination was unremarkable, and no features suggestive of postoperative complications were identified. No follow-up imaging was performed in view of the patient’s satisfactory clinical progress.

## Discussion

Foreign body ingestion remains a significant pediatric health concern, particularly in young children, due to their natural tendency for oral exploration. While most small blunt objects pass spontaneously through the gastrointestinal tract, clinical outcomes are primarily determined by the object’s physical characteristics and the site of impaction, which influence the risk of complications such as obstruction, perforation, and fistula formation [1,2].

Among these, magnet ingestion represents a particularly high-risk subset, especially with the increasing availability of high-powered rare-earth magnets in toys and household items [3-5]. Neodymium-iron-boron magnets generate strong attractive forces even across intervening bowel loops, leading to sustained tissue compression. This mechanism predisposes to progressive ischemia, pressure necrosis, and eventual transmural injury.

Clinical presentation is frequently nonspecific and may include vomiting, abdominal pain, constipation, sore throat, or signs of peritonitis in advanced cases [6,7]. Because ingestion is often unwitnessed, diagnosis may be delayed, increasing the risk of serious complications. In contrast to single magnet ingestion, which may sometimes be managed conservatively in selected asymptomatic patients, ingestion of multiple magnets is associated with a high rate of bowel injury and often requires operative intervention [8,9].

Radiologic evaluation plays a central role in diagnosis and management. Plain abdominal radiography can identify most ingested foreign bodies and provides useful information regarding the number, configuration, and location [7]. Observation may be appropriate for a single blunt magnet in an asymptomatic child without co-ingestion of another metallic object or clinical signs of peritoneal irritation [10]. However, multiple magnetic beads may align in a chain-like pattern, making it difficult to distinguish a single intraluminal cluster from magnets lodged in separate bowel segments [8,9]. Symptoms may also be delayed, with some patients presenting days to weeks after ingestion.

Management of magnet ingestion is guided mainly by consensus recommendations and institutional algorithms rather than a single standardized protocol [11,12]. Endoscopic retrieval is recommended when magnets are located in the esophagus or stomach, whereas surgical intervention is often necessary when multiple magnets have passed beyond the pylorus or when there is evidence of fixation, obstruction, or peritoneal irritation.

In the present case, several factors likely contributed to the development of a gastrocolic fistula. The ingestion was unwitnessed, and early symptoms were nonspecific, delaying presentation. Radiographic appearance of clustered beads may have underestimated the presence of magnets across adjacent bowel segments. Endoscopic retrieval was incomplete, possibly due to partial embedding of magnets within the inflamed mucosa and their fixation across the gastric and colonic walls, limiting safe extraction. Continued inter-bowel magnetic attraction likely resulted in progressive pressure necrosis and eventual fistula formation between the posterior gastric antrum and transverse colon.

Only limited published reports describe gastrocolic fistula following pediatric magnet ingestion. In previously reported cases, children were also young, diagnosis was delayed, and surgery was ultimately required after severe transmural gastrointestinal injury [13,14]. Compared with those reports, the present case similarly demonstrates delayed recognition and fistula formation. The delay in diagnosis has been consistently associated with greater morbidity, including perforation, fistula formation, and bowel obstruction [13]. This case highlights the unpredictable injury pattern associated with magnetic foreign bodies and underscores the importance of early recognition, serial imaging, and timely multidisciplinary management to prevent further gastrointestinal injury [14].

## Conclusions

Multiple magnet ingestion in children may result in severe transmural gastrointestinal injury. In this case, unwitnessed ingestion and nonspecific early symptoms contributed to a delay in diagnosis, while incomplete endoscopic retrieval necessitated further intervention. The development of a gastrocolic fistula highlights the risk of transmural injury and the limitations of endoscopic management in advanced cases. This case underscores the importance of early suspicion, complete retrieval, and timely surgical escalation when transmural injury is suspected. Strengthening parental awareness and preventive measures may help reduce the occurrence of such cases.
